# Insulin Action, Glucose Homeostasis and Free Fatty Acid Metabolism: Insights From a Novel Model

**DOI:** 10.3389/fendo.2021.625701

**Published:** 2021-03-16

**Authors:** Darko Stefanovski, Naresh M. Punjabi, Raymond C. Boston, Richard M. Watanabe

**Affiliations:** ^1^ School of Veterinary Medicine, University of Pennsylvania, New Bolton Center, PA, United States; ^2^ Division of Pulmonary and Critical Care Medicine, Johns Hopkins University School of Medicine, Baltimore, MD, United States; ^3^ Department of Preventive Medicine, Keck School of Medicine of USC, Los Angeles, CA, United States

**Keywords:** free fatty acids (FFA), insulin action, FFA metabolism, glucose, lipolysis

## Abstract

Glucose and free fatty acids (FFA) are essential nutrients that are both partly regulated by insulin. Impaired insulin secretion and insulin resistance are hallmarks of aberrant glucose disposal, and type 2 diabetes (T2DM). In the current study, a novel model of FFA kinetics is proposed to estimate the role insulin action on FFA lipolysis and oxidation allowing estimation of adipose tissue insulin sensitivity (*S_IFFA_*). Twenty-five normal volunteers were recruited for the current study. To participate, volunteers had to be less than 40 years of age and have a body mass index (BMI) < 30 kg/m^2^, and be free of medical comorbidity. The proposed model of FFA kinetics was used to analyze the data derived from the insulin-modified FSIGT. Mean fractional standard deviations of the parameter estimates were all less than 20%. Standardized residuals of the fit of the model to the FFA temporal data were randomly distributed, with only one estimated point lying outside the 2-standard deviation range, suggesting an acceptable fit of the model to the FFA data. The current study describes a novel one-compartment non-linear model of FFA kinetics during an FSIGT that provides an FFA metabolism insulin sensitivity parameter (*S_IFFA_*). Furthermore, the models suggest a new role of glucose as the modulator of FFA disposal. Estimates of *S_IFFA_* confirmed previous findings that FFA metabolism is more sensitive to changes in insulin than glucose metabolism. Novel derived indices of insulin sensitivity of FFA (*S_IFFA_*) were correlated with minimal model indices. These associations suggest a cooperative rather than competitive interplay between the two primary nutrients (glucose and FFA) and allude to the FFA acting as the buffer, such that glucose homeostasis is maintained.

## Introduction

Glucose and free fatty acids (FFA) are essential nutrients that are both partly regulated by insulin. While insulin’s role in glucose metabolism promotes disposal in peripheral tissues such as muscle, insulin action in the adipose tissue, mainly suppresses lipolysis. In individuals with obesity or type 2 diabetes suffer from insulin resistance, a state where insulin is inefficient in performing the above outlined roles (1). Furthermore, there is evidence that many of the adverse metabolic effects of glucose intolerance, such as insulin resistance and type 2 diabetes, may be mediated by FFA and have termed lipotoxicity (2). It has been proposed that type 2 diabetes is a consequence of aberrant lipid metabolism (2–4), which supports the concept of interaction between insulin, glucose and FFA homeostasis. Hence, identifying methods that combine simple experimental protocols that yield data that can be used to estimate indices of insulin sensitivity on the level of adipose tissue is of great importance. Furthermore, it will be beneficial if these strategies simultaneously quantify the interaction between glucose and FFAs.

There is no agreement on the best methodology for estimating insulin sensitivity (5). Previous approaches that estimate adipocyte level insulin sensitivity can be loosely divided into 3 classes. First, previously a method has been developed that uses simple experimental data (postabsorptive FFA and insulin) and simple calculations adipose tissue insulin resistance index [Adipo-IR (6)]. This method is the analog of the previously developed index of homeostatic model assessment (HOMA) of whole-body glucose insulin resistance [HOMA-IR (7)]. One potential problem with this model is that while fasting glucose is maintained within a narrow range by a feedback loop mechanism involving insulin, no such mechanism is known relating to FFA homeostasis (5). Thus, Adipo-IR may be a less reliable estimate of adipose insulin resistance. Second, the multistep pancreatic clamp experimental protocol provides methodology for estimating adipose tissue insulin sensitivity, whereby using somatostatin to keep endogenous insulin concentration fully suppressed, the exogenously imposed insulin concentration that provides 50% suppression of lipolysis (IC_50_) yields the desired index of adipose tissue insulin resistance. However, the complexity and the time involved in performing the multistep pancreatic clamp makes its adaptation difficult (8). Third and final are the methodologies that use simple experimental protocols such as the frequently sampled intravenous glucose tolerance test (FSIGTT) or the oral glucose tolerance test (OGTT) and sophisticated mathematical models that based on the data provide estimates of indices of insulin sensitivity. While these models have been found to be based on experimental data that is more physiologically plausible, they rely on set of simplifying assumptions regarding the kinetics of the system which may not be fully validated to approaches that do not rely on such underlying assumptions (5). The methodology presented here belongs to the third class of approaches of estimating adipose tissue insulin sensitivity.

Previously, several models of FFA kinetics have been proposed that address the bidirectional interaction between insulin and FFA either directly or indirectly (9–15). Many of these available models are limited in that they have complex mechanisms requiring multiple parameters with assumed values, are dependent upon experimental protocols not commonly utilized in the clinical setting (9), are based on underlying assumptions that and not based on observations (11), or only partially use the available data (10, 12). In contrast to previous models, our novel model of FFA kinetics was specifically designed to provide quantitative measures of sensitivity of FFA to the actions of insulin and oxidation allowing estimation of insulin sensitivity on FFA metabolism (*S_IFFA_*). Another unique feature of our novel model is that it estimates the contribution of plasma glucose as a regulator of FFA oxidation. Estimates derived from the novel model of FFA kinetics are compared with other model-based approaches and with previously published experimental parameters of FFA metabolism.

## Methods

### Model Development

The primary objective was to develop a parsimonious model (16) that would characterize plasma FFA kinetics during a frequently sample intravenous glucose tolerance test (FSIGT). The following assumptions were made in the development of the model: (a) plasma insulin does not directly influence FFA kinetics, but acts through a “remote” compartment to exert its action; (b) insulin action controls the rate of lipolysis; (c) plasma glucose regulates FFA disposal in proportional manner; (d) suppression of FFA is at its maximum at the time of the insulin bolus at 20 minutes. The model acceptance criteria were that, on per subject basis, the model will accurately recreate the FFA time profile where the standardized residuals do not exhibit any systemic deviation and are within the range of two standard deviations. Furthermore, all parameters of the proposed model had to be uniquely identified with fractional standard deviations (FSD) below 0.5.

### Mathematical Model of FFA Kinetics During an FSIGT Test

**Figure 1:**
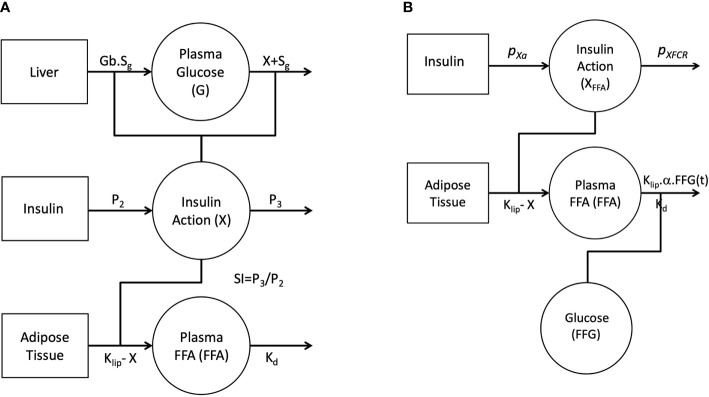
Graphical depiction of the initial single-pool non-linear models of Glucose and FFA **(A)**. Insulin in the remote compartment (*X*, insulin action with units 1/min) regulates the appearance and elimination of plasma glucose and FFA lipolysis rate. Final non-linear single pool FFA model where insulin action on FFA (*X_FFA_*, units 1/min) modulates the rate of lipolysis and plasma glucose (*FFG*, units mg/dl) regulates the disposal of FFA **(B)**.

**Figure 1A** depicts the full FFA model, which was then reduced. The proposed model of plasma FFA kinetics during an FSIGT (**Figure 1B**) is described with the following set of differential equations.


(1) $$\frac{dFFA}{dt}=-\left(S_{FFA}\cdot \alpha \cdot FFG(t)\right)\cdot FFA(t)+\left(S_{FFA}-X_{FFA}(t)\right)\cdot FFA_{b};\;FFA(0)=FFA_{b}$$

(2) $$\frac{dX_{FFA}}{dt}=-P_{XFCR}\cdot X_{FFA}\left(t\right)+P_{X\alpha }\cdot \left(I\left(t\right)-I_{b}\right);X_{FFA}\left(0\right)=0$$

Where *FFG(t)* is linear interpolation of glucose plasma concentration at time *t*. *S_FFA_* is the fractional FFA disposal rate with units 1/min. Parameter α serves dual purpose as a unit conversion and scaling factor for the effect of plasma glucose, *FFG(t)*, on the disposal of FFA with units 1/mmol/l. It is assumed that the FFA plasma concentration at time 0, *FFA(0)*, is equal to the fasting FFA concentration, *FFA_b_*.

Plasma FFA kinetics (*FFA(t)*) are linked to insulin action on FFA (*X_FFA_(t)*) kinetics and glucose homeostasis *via* equation 1. Insulin action is defined in equation 2, where plasma insulin increment above basal (I(t) – Ib) contributes to insulin action similar to minimal model (16) and we assume insulin action at steady-state is equal to zero. Parameters *P_XFCR_* and *P_Xa_* are also analogous to the *P_2_* and *P_3_* parameters of the minimal model. Parameter *P_XFCR_* is the fractional disposal rate of insulin from the remote compartment with units 1/min. The parameter *P_Xa_* also serves a dual function as a unit conversion and a fractional transfer rate with units 1/min^2^.mU/l. The Adipose Tissue insulin sensitivity parameter (*SI_FFA_*) is calculated as the ratio between *P_XFCR_* and *P_Xa_*,


(3) $${\mathrm{SI}}_{FFA}=\frac{P_{XFCR}}{P_{X_{\alpha }}}$$

### Study Sample

Normal healthy volunteers were recruited from the local Baltimore-Washington area as previously outlined (**Table 1**) (17). Briefly, after institutional review board (IRB) study approval, eligibility for participation required a BMI < 30 kg/m^2^ and absence of the following physician diagnosed conditions: type 2 diabetes mellitus, angina, myocardial infarction, coronary revascularization, congestive heart failure, stroke, obstructive lung disease, renal or hepatic dysfunction, or neurologic disease. After an initial telephone screening, eligible volunteers were required to complete a serologic screen to confirm absence of an abnormal fasting glucose. Informed consent was obtained from each volunteer and the study protocol was approved by the local institutional review board.

**Table 1:** Mean ± SE subject characteristics (n = 25).

Age (years)	25.2 ± 4.7
BMI (kg/m^2^)	23.6 ± 2.7
Fasting Glucose [mM]	5.25 ± 0.07
Fasting Insulin [pM]	45.11 ± 3.13
Free Fatty Acids [μM]	325.84 ± 23.5

### Study Protocol

The FSIGT was performed as previously described (13). An intravenous line was placed in the right and left antecubital veins for blood sampling and kept patent with a continuous infusion of 0.9% saline. The intravenous line in the dominant arm was used for blood sampling while glucose and insulin were administered through the contralateral intravenous line. Basal sampling occurred at −15, −10, −5, and −1 min before glucose administration. Glucose (50% dextrose, 0.3 g/kg) was administered intravenously at time zero over one minute followed by infusion of normal saline. Twenty minutes after the glucose injection, regular insulin (0.03 U/kg) was injected. Blood samples were collected at 2, 3, 4, 5, 6, 8, 10, 12, 14, 16, 19, 22, 24, 25, 27, 30, 40, 50, 60, 70, 80, 90, 100, 120, 140, 160, and 180 min post-glucose injection.

Glucose was measured enzymatically in duplicate using a Glucose Analyzer II (Beckman Instruments, Fullerton, CA). Insulin concentrations were determined in duplicate by radioimmunoassay using standard commercial kits (Linco Research; St Charles, MO). Free fatty acids (NEFA C, Wako Pure Chemical Industries; Richmond, VA, USA) were measured using colorimetric methods in commercially available kits.

### Statistical Analysis

Model parameter estimation was performed using WinSAAM (University of Pennsylvania, Kennett Square, PA), which uses modified Chu-Berman numerical integrator for solving the model equations and a variant of the generalized non-linear weighted least squares version of the Gauss-Newton optimizer (18). Weights were computed as the inverse variance from the FFA assay and invoked in the data fitting step as fractional standard deviations of 5%. Parameter estimates of the mathematical model were obtained by fitting (obtaining point estimates) FFA estimated temporal profile to the observed FFA concentrations for each individual subject.

Statistical analysis was performed using Stata 15MP (StataCorp, College Station TX). All descriptive statistics of the observed data are shown as mean ± SEM unless otherwise stated. Normality testing was used to assess the skweness of the data. Correlation analysis was performed using Spearman rank correlation. In order to establish equivalence between *X* and *X_FFA_*, t-test was used to assess the similarity between the peaks in the insulin actions. All findings were deemed significant at the level of α=0.05 for the probability of a Type I error.

## Results

### Kinetic Analysis of Insulin Modified FSIGT

It was initially assumed that insulin action (X(t)) was identical for both glucose and FFA and a model was constructed incorporating this feature (**Figure 1A**). Similar to minimal model, the model of FFA kinetics was developed as a non-linear model of FFA kinetics during FSIGT. As graphically depicted in **Figure 1A**, it was assumed the changes in insulin action were due to change in plasma glucose and FFA during an FSIGT. In the novel FFA model, Insulin Action (X) modulated the suppression of lipolysis in adipose tissue. However, when all six parameters of the model were simultaneously fitted using FSIGT data from the 25 healthy volunteers, parameter estimates were poor with FSDs exceeding 50% and the glucose and insulin time predicted profiles showed significant systematic deviations from the data. These results suggest that the mechanism of insulin action is likely different for FFA compared to glucose. We made two significant changes to the model of FFA in order to improve the fit of the model to the observed data. First, we formulate a novel mathematical construct termed FFA insulin action (*X_FFA_*). Analogues to insulin action, *X_FFA_* arises as a result of changes in plasma FFA concentration alone and regulates the suppression of lipolysis, **Figure 1B**. Second and final, the plasma FFA rather than being estimated independent of glucose, is a subject to being controlled by it (glucose is another input to the new model) *via* the suppression of FFA disposal.

**Figure 2:**
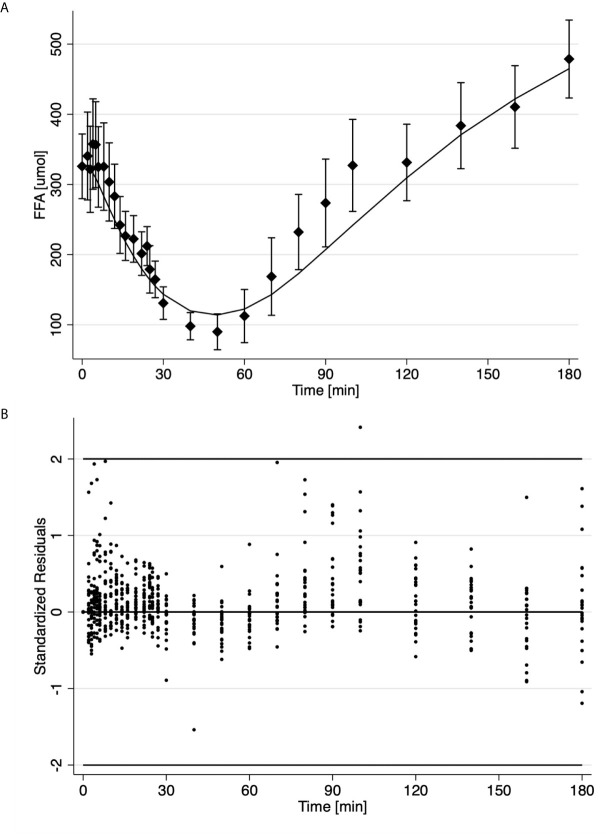
Time course of the average ± SE (solid diamonds) and estimates (solid line) of FFA data **(A)**; Standardized residuals where each dot is one observation from different subject **(B)**.

Results for the analysis of the FSIGT data on the 25 health volunteers using the final model of FFA kinetics is shown in **Table 2** and **Figure 2**. **Figure 2A** illustrates the fit of the model to the average temporal profile of plasma FFA concentration. It can be seen that in the period of 45 to 100 minutes, there is a modest systemic deviation of the model from the observed data. The average temporal profile of plasma FFA suggests a faster increase plasma concentration than the one suggested by the model followed by phase (100 to the end of the experiment) with slower rate of increase in FFA plasma levels. Upon visual inspection of the individual FFA temporal profiles (data not shown) it was established that this was due to a subset of observation in the above-mentioned interval present in 4 subjects (16%). Nevertheless, standardized residuals of the fit of the model to the FFA temporal data appeared randomly distributed, with only one estimated point lying outside the 2-standard deviation range, suggesting an acceptable fit of the model to the FFA data (**Figure 2B**). Mean fractional standard deviations of the parameter estimates were all less than 20% (**Table 2**), consistent with the model being identifiable from the data.

**Table 2:** Mean ± SE parameter estimates (n=25).

Parameter	Value ± SE	Units	FSD ± SE
α	1.0E-01 ± 1.0E-02	mmol/l^−1.^min^−1^	0.09 ± 0.02
*S_FFA_*	2.0E-02 ± 2.0E-03	min^−1^	0.11 ± 0.01
*p_XFCR_*	6.0E-02 ± 1.0E-02	min^−1^	0.17 ± 0.02
*p_Xa_*	3.4E-05 ± 1.1E-05	pmol/l^−1.^ min^−2^	0.08 ± 0.01
*SI*_FFA_	5.3E+00 ± 7.8E-01	10^−4.^pmo1/1^−1.^ min^−1^	0.11 ± 0.02

The associations between parameter estimates from the proposed FFA model and metabolic indices from the traditional minimal model were assessed (**Table 3**) to examine similarities of the underlying mechanisms quantifying the various indices of the model. These results showed that the *S_I_* from the minimal model was moderately correlated with *S_IFFA_* (ρ=0.53, P=0.006, **Table 3**). There was also a strong inverse correlation between acute insulin response to glucose (*AIRg*) estimated by the minimal model and *S_IFFA_* (ρ = −0.76, P≤0.0001, **Table 3**). Not surprisingly, the *DI* estimated from the minimal model, product of *AIRg* and *S_I_*, was also correlated with *S_IFFA_* (ρ = −0.55, P=0.005, **Table 3**). The observed correlations of *S_IFFA_* with various indices of the minimal model were probably due to their observed association with *p_Xa_* (**Table 3**) because *S_IFFA_* is calculated as the ratio between *p_Xa_* and *p_XFCR_* parameters (analogs to the original minimal model parameters *P_3_* and *P_2_*). No additional significant correlations were observed between minimal model indices and parameters of our novel FFA model.

**Table 3:** Spearman’s rank correlation between MINMOD and novel FFA model parameter estimates (n=25).

	α	*S_FFA_*	*p_XFCR_*	*p_Xa_*	*SI_FFA_*
***S_I_***	−0.01	−0.06	0.16	**0.52**	**0.53**
***P***	0.965	0.773	0.434	**0.007**	**0.006**
*** ***					
***Sg***	−0.23	−0.25	0.21	−0.12	−0.23
***P***	0.272	0.236	0.312	0.580	0.265
*** ***					
***AIRg***	0.04	−0.15	−0.35	−**0.84**	−**0.76**
***P***	0.861	0.473	0.083	**≤0.0001**	**≤0.0001**
*** ***					
***DI***	0.01	−0.16	−0.08	−**0.57**	−**0.55**
***P***	0.962	0.447	0.712	**0.003**	**0.005**
*** ***					
***G_0_***	−0.14	−0.17	0.03	−0.12	−0.18
***P***	0.493	0.408	0.884	0.570	0.400
*** ***					
***p_2_***	−0.06	0.07	0.35	0.34	0.06
***P***	0.787	0.735	0.082	0.098	0.776
*** ***					
***p_3_***	−.04	−0.05	0.28	**0.51**	0.38
***P***	0.864	0.808	0.170	**0.010**	0.063

Bolded values are statistically significant (P<0.05).

**Figure 3:**
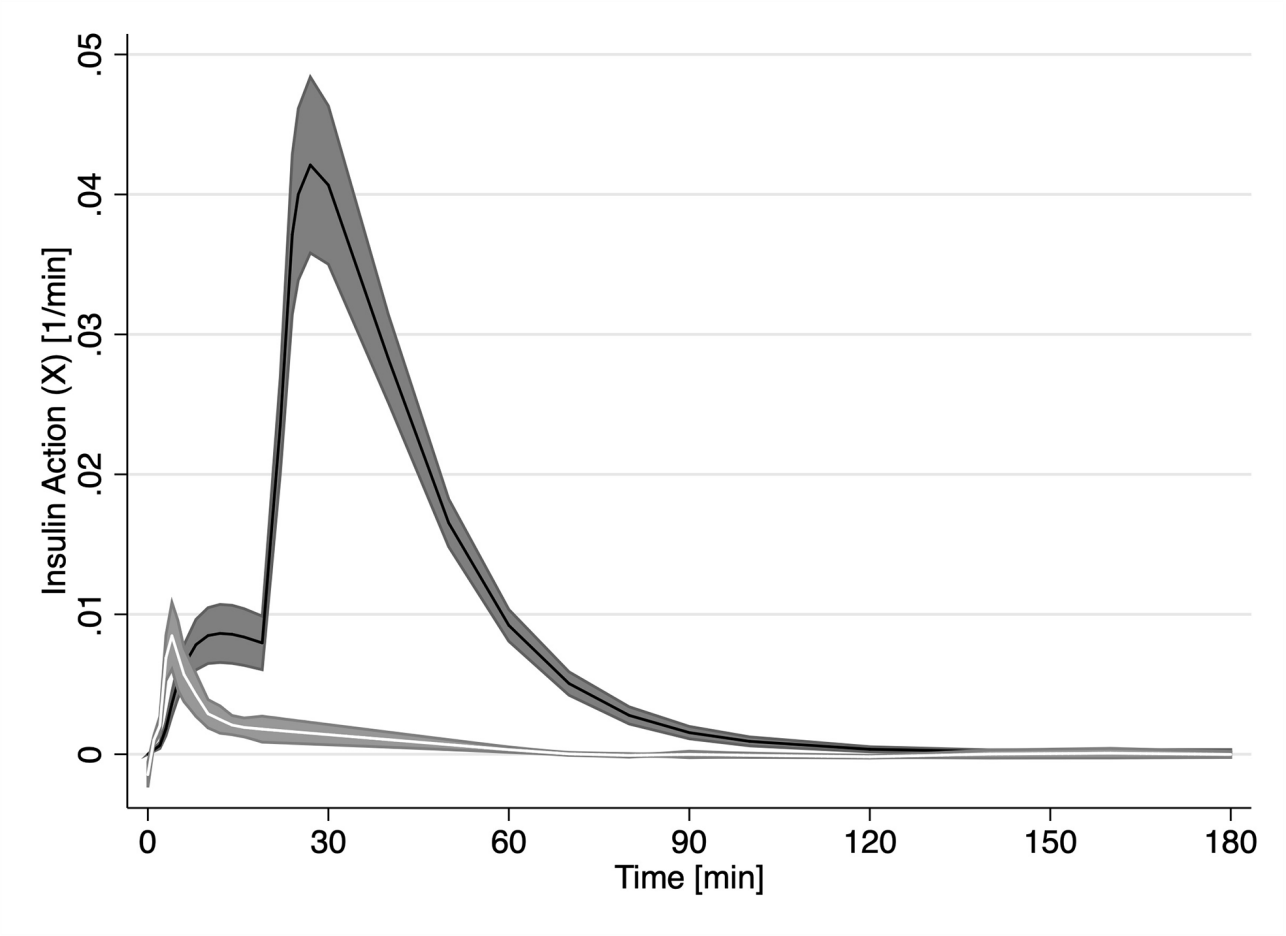
Time course of estimated insulin action (X, solid black line) estimated by the minimal model and FFA insulin action (X_FFA_, solid white line) from our novel model of FFA kinetics. Grey areas represent the SEM.

While *X_FFA_* and *X* are analogues to their model specification, **Figure 3** reveals major difference between the two. On average, FFAs first experience the effect of insulin action (*X_FFA_*) at approximately 4 min post challenge. Endogenous glucose insulin action averaged (*X*, first smaller peak in **Figure 3**) peaked on average at 12 minutes. Interestingly, there was no significant difference between the magnitude of the two insulin actions (Peak insulin action on glucose, X_max_ was not significantly different from Peak insulin action on FFA, X_maxFFA,_ (0.0086 min^−1^, p > 0.05). The profile of *X_FFA_* closely resembled the profile of plasma Insulin in the first 10 minutes.

## Discussion

Since the seminal work by Randle et al. (19), there has been a bias towards casting FFAs as “metabolic villains” (20). In fact, previous work has shown that acute elevation in plasma FFA leads to impaired hepatic gluconeogenesis and overall decreased glucose tolerance (21–24). Increased plasma FFA is also associated with reduced hepatic insulin clearance (25). Furthermore, intravenous intralipid/heparin infusions result in increased intramyocellular lipid, which has been related to insulin resistance. These observations highlight the various metabolic aberrations associated with increased supply of FFA (26).

Recently it has been recognized that impaired FFA disposal may be as important in the accumulation of fat in non-adipose tissue increased FFA uptake (27). By contrast, other studies suggest increased plasma FFA are associated with compensatory insulin secretion responsible for maintaining almost unchanged glucose tolerance in the face of increasing insulin resistance (28–30). Finally, it has been proposed that type 2 diabetes perhaps results from aberrant lipid metabolism (2–4). In animal models, it has been shown that obesity, which is often associated with chronically elevated levels of insulin, leads to decreased FFA oxidation in the resting state (31). Thus, to delineate the role of FFA in glucose metabolism and glucose control over FFA homeostasis will indeed require a better understanding of insulin’s influence on FFA metabolism.

The FSIGT is a widely-accepted approach for assessing glucose homeostasis that does not require the use of tracers. The purpose of the novel FFA model was to extend the usability of the FSIGT experimental approach so it provides a more comprehensive metabolic picture. Recently, the insulin-modified FSIGT has been used to study the kinetics of plasma FFA (13). The current study reveals that the plasma FFAs have very rich dynamic highly amenable to mathematical modeling. Previously, several models that explain the time course of FFA during an FSIGT have been developed (10–12, 32). The model by Thomaseth and Pavian (6) attempts to explain the profile of plasma FFA during the FSIGT. One of the features of their model is that the plasma FFA at the end of the FSIGT returns to pre-glucose injection levels (11). The latter assumption severely hinders the usability of their model, because it has been well established that plasma FFA levels at the end of the FSIGT may exceed the basal FFA concentrations by as much as 50% due to a rebound effect (13). Furthermore, and as we stated in the introduction, while glucose is under a strong feedback loop control, no such mechanism has been established for FFA (5). Hence any mathematical model that tends to accurately represent FFA kinetics must provide a formulation that permits for different equilibrium point from the assumed starting equilibrium. In contrast, models developed by Roy and Parker (17) and Periwal and colleagues (22) make no assumptions regarding the final FFA concentrations. However, neither of these models utilize measurements from the last 60 minutes of the FSIGT, presumably because they cannot estimate the data during this interval (12). Our novel model is also capable of resolving the full temporal profile of plasma FFA regardless of the final concentration of FFAs. Furthermore, in simulation studies not shown here we observed that the model was capable of reaching a new equilibrium state and thus indicating that our novel mathematical model is stable. Interestingly, all three models previously mentioned use glucose, FFA and insulin data to simultaneously estimate both glucose and FFA. The model by Boston and Moate departs from this paradigm and utilizes only the glucose to resolve the profile of plasma FFA (32). In their model, glucose reaches a “remote” compartment *via* a time delay before it exerts its action on FFA lipolysis. This “remote” compartment is considered to be either a proxy for the action of insulin on lipolysis or the direct effect of elevated glucose levels in the “remote” compartment on the rate of lipolysis (33). Thus, their model assumes that any impairment of glucose metabolism will concordantly impact FFA metabolism. Nevertheless, the model by Boston and Moate was not intended to quantify the effect of insulin such as insulin sensitivity of FFA metabolism (*S_IFFA_*).

The model of FFA kinetics during an FSIGT proposed herein was based on three simplifying assumptions. First, insulin does not directly influence FFA kinetics. Identical to the concept of the remote insulin effect (16), it was assumed that insulin had to survive transcapillary transport, which is the rate-limiting step for insulin action, to exert its effects on FFA kinetics. Insulin can take up to 20 min to traverse the transendothelial space and exert its effect on glucose kinetics (34). This time corresponded well with previously identified first phase in the plasma FFA time profile (also known as the plateau) during which there is no noticeable change in the plasma FFA concentration (13). Furthermore, this period also corresponded well to the time delay parameter, τ, in the model by Boston and Moate (32). Second, a new set of parameters were defined for insulin action on FFA based on the framework for remote insulin action from the minimal model and estimated independently of insulin action on glucose (see Equations 1 and 2, and **Figure 3**). The notion that insulin action has different kinetics for FFA is not new. Jensen and colleagues have shown that the suppression of FFA lipolysis *via* HSL is extremely sensitive to insulin (35). Furthermore, two other models of FFA kinetics also define different actions of insulin on FFA and glucose (10, 12). Third, insulin influences the suppression of FFA lipolysis, while glucose controls FFA oxidation. Previous models have assumed that FFA disappearance from plasma is mainly driven by decreased lipolysis, while FFA oxidation remains constant (12, 32). The mathematical formulation of the FFA model presented in the current study implies that FFA utilization is under a direct and proportional control of glucose. Previously, it has been shown that when carbohydrates are in abundance, the liver does not only primarily utilize glucose but also converts it to FFA. In hepatocytes, FFAs are readily esterified with glycerol 3-phosphate to generate TAG or combined with cholesterol to produce cholesterol esters (36). Because of the enhanced hepatic FFA metabolism, plasma FFA concentration falls. While much of the literature has been dedicated to the competitive nature of the association between FFA and glucose, our formulation embraces the concept of a coordinated nature of the association between these two substrates previously reported in human muscle (37). Fourth, the insulin administered during the insulin-modified FSIGT has no influence on FFA disposal. Porte and colleagues have shown the additional insulin dose is above the threshold of activation for extra receptors and hence does not play a significant role in insulin-dependent FFA disposal (38). Sumner and colleagues have shown the multiphasic response of FFA during an FSIGT is non-responsive to exogenous insulin (13). Additionally, Jensen and colleagues have shown that the difference in insulin action on lipolysis between obese, insulin resistant, and insulin sensitive is not in the rate at which lipolysis is suppressed, but more at the level of suppression suggesting that insulin suppression of lipolysis is saturable process (35). Therefore, it is highly likely that by the time the insulin bolus takes its full effect in the remote compartment, lipolysis is already maximally suppressed.

Comparing the estimates of our model parameters to previously published estimates show that our estimates were consistently smaller. Previous studies that utilized isotopic tracer to estimate endogenous lipolysis rate report a rate of 3.13 μmol/kg/min compared to our estimate of average lipolysis of 1.24 ± 0.14 μmol/kg/min for our cohort (39). Horowitz and colleagues determined FFA oxidation to be between 1.13 and 1.6 μmol/kg/min using isotopic tracer methods, which is higher compared to our average estimate of 0.72 ± 0.14 μmol/kg/min (39). Contrasting the literature-derived values of lipolysis and FFA oxidation show almost 2:1 relationship respectively between these rates, which is precisely the relationship of our estimates of lipolysis and FFA disposal. One possible reason for the discrepancy may be that our cohort consisted of healthy young volunteers which is in marked contrast to other studies that have enrolled older individuals. Nevertheless, it is encouraging that the ratio between lipolysis and FFA oxidation was similar to what has been previously observed.

The statistically significant correlations in **Table 3** indicate that *S_IFFA_* is associated with all the minmod indices through *p_Xa_*. From the specification of the model in **Figure 1B**, *p_Xa_* is defined as being the index of the rate of appearance of insulin in the remote compartment, insulin action. It has been previously shown that insulin resistance is associated with decreased trans-endothelial transport (40). Therefore, it appears that the restricted access of insulin to the interstitial space is also limiting the supply of insulin required to suppress FFA lipolysis. Interestingly, *p_Xa_* is also inversely correlated to *DI*. It is worth noting that we have not observed the same trend with *p_3_* from the minimal model. The association between *DI* and *p_Xa_* may indicate that as the glucose tolerance increases, the fraction of insulin partitioned as *X_FFA_* is decreasing. As such it only emphasizes the role of coordinated metabolism between FFA and glucose where FFA serves as a buffer fuel absorbing and dampening disturbances in glucose metabolism to promote stable glucose homeostasis. Future studies will be required to quantify more precisely this relationship.

In conclusion, the current study describes a novel one-compartment non-linear model of FFA kinetics during an FSIGT that, for the first time, provides an FFA metabolism insulin sensitivity parameter (*S_IFFA_*). Estimates of *S_IFFA_* confirmed previous findings that FFA metabolism is more sensitive to changes in insulin than glucose metabolism. Novel derived indices of insulin sensitivity of FFA (*S_IFFA_*) were correlated with previous Bergman’s minimal model indices. These associations propose a cooperative rather than competitive relationship between the two primary nutrients (glucose and FFA) and allude to the FFA acting as the buffer, such that glucose homeostasis is maintained. The new model proposed in this study is likely to shed useful insights into the changes in FFA metabolism during development of insulin resistance and type 2 diabetes.

## Data Availability Statement

The raw data supporting the conclusions of this article will be made available by the authors, without undue reservation.

## Ethics Statement

The studies involving human participants were reviewed and approved by IRB Johns Hopkins University. The patients/participants provided their written informed consent to participate in this study.

## Author Contributions

DS contributed to the study concept, analyzed the data, and drafted the manuscript. NP contributed to the study design, collected the data, drafted and reviewed the manuscript. RB contributed to the mathematical modeling concepts, statistical analysis, drafted and reviewed the manuscript. RW contributed to the study concept, analyzed the data, drafted and reviewed the manuscript. DS is the guarantor of this work and, as such, had full access to all the data in the study and takes responsibility for the integrity of the data and the accuracy of the data analysis. All authors contributed to the article and approved the submitted version.

## Funding

NP has received grant support from the National Institutes of Health (HL075078 and HL117167) for this work.

## Conflict of Interest

The authors declare that the research was conducted in the absence of any commercial or financial relationships that could be construed as a potential conflict of interest.
