# Corrigendum: Insulin Action, Glucose Homeostasis and Free Fatty Acid Metabolism: Insights From a Novel Model

**DOI:** 10.3389/fendo.2021.789390

**Published:** 2021-10-27

**Authors:** Darko Stefanovski, Naresh M. Punjabi, Raymond C. Boston, Richard M. Watanabe

**Affiliations:** ^1^ School of Veterinary Medicine, University of Pennsylvania, New Bolton Center, PA, United States; ^2^ Division of Pulmonary and Critical Care Medicine, Johns Hopkins University School of Medicine, Baltimore, MD, United States; ^3^ Department of Preventive Medicine, Keck School of Medicine of USC, Los Angeles, CA, United States

**Keywords:** free fatty acids (FFA), insulin action, FFA metabolism, glucose, lipolysis

In the original article, there was a mistake in **Table 2** as published. **The units for parameter α and *SI_FFA_***. The corrected **Table 2** appears below.

**Table 2:** Mean ± SE parameter estimates (n=25).

Parameter	Value ± SE	Units	FSD ± SE
α	1.0E-01 ± 1.0E-02	mmol/l^-1^	0.09 ± 0.02
*S_FFA_*	2.0E-02 ± 2.0E-03	min^-1^	0.11 ± 0.01
*p_XFCR_*	6.0E-02 ± 1.0E-02	min^-1^	0.17 ± 0.02
*p_Xa_*	3.4E-05 ± 1.1E-05	pmol/l^-1^.min^-2^	0.08 ± 0.01
*SI*_FFA_	5.3E+00 ± 7.8E-01	10^-4^.pmol/l^-1^.min^-1^	0.11 ± 0.02

The authors apologize for this error and state that this does not change the scientific conclusions of the article in any way. The original article has been updated.

## Publisher’s Note

All claims expressed in this article are solely those of the authors and do not necessarily represent those of their affiliated organizations, or those of the publisher, the editors and the reviewers. Any product that may be evaluated in this article, or claim that may be made by its manufacturer, is not guaranteed or endorsed by the publisher.

